# Subnormothermic Perfusion with H_2_S Donor AP39 Improves DCD Porcine Renal Graft Outcomes in an Ex Vivo Model of Kidney Preservation and Reperfusion

**DOI:** 10.3390/biom11030446

**Published:** 2021-03-17

**Authors:** Smriti Juriasingani, Aushanth Ruthirakanthan, Mahms Richard-Mohamed, Masoud Akbari, Shahid Aquil, Sanjay Patel, Rafid Al-Ogaili, Matthew Whiteman, Patrick Luke, Alp Sener

**Affiliations:** 1 Department of Microbiology and Immunology, University of Western Ontario, London, ON N6A 3K7, Canada; sjuriasi@uwo.ca (S.J.); vetman@gmail.com (M.A.); patrick.luke@lhsc.on.ca (P.L.); 2 Matthew Mailing Center for Translational Transplant Studies, London Health Sciences Center, London, ON N6A 5A5, Canada; aruthira@uwo.ca (A.R.); Mahmoud.Richard-Mohamed@lhsc.on.ca (M.R.-M.); shahid.aquil@lhsc.on.ca (S.A.); sanjay.patel@lhsc.on.ca (S.P.); rafid.alogaili@lhsc.on.ca (R.A.-O.); 3 Multi-Organ Transplant Program, London Health Sciences Center, London, ON N6A 5A5, Canada; 4 University of Exeter Medical School, St. Luke’s Campus, Exeter EX1 2LU, UK; M.Whiteman@exeter.ac.uk; 5 Department of Surgery, London Health Sciences Center, London, ON N6A 5A5, Canada

**Keywords:** AP39, hydrogen sulfide, subnormothermic perfusion, kidney preservation, ischemia–reperfusion injury, donation after cardiac death, porcine model

## Abstract

Cold preservation is the standard of care for renal grafts. However, research on alternatives like perfusion at higher temperatures and supplementing preservation solutions with hydrogen sulfide (H_2_S) has gained momentum. In this study, we investigated whether adding H_2_S donor AP39 to porcine blood during subnormothermic perfusion at 21 °C improves renal graft outcomes. Porcine kidneys were nephrectomized after 30 min of clamping the renal pedicles and treated to 4 h of static cold storage (SCS) on ice or ex vivo subnormothermic perfusion at 21 °C with autologous blood alone (SNT) or with AP39 (SNTAP). All kidneys were reperfused ex vivo with autologous blood at 37 °C for 4 h. Urine output, histopathology and RNAseq were used to evaluate the renal graft function, injury and gene expression profiles, respectively. The SNTAP group exhibited significantly higher urine output than other groups during preservation and reperfusion, along with significantly lower apoptotic injury compared to the SCS group. The SNTAP group also exhibited differential pro-survival gene expression patterns compared to the SCS (downregulation of pro-apoptotic genes) and SNT (downregulation of hypoxia response genes) groups. Subnormothermic perfusion at 21 °C with H_2_S-supplemented blood improves renal graft outcomes. Further research is needed to facilitate the clinical translation of this approach.

## 1. Introduction

Over the past 25 years, hydrogen sulfide (H_2_S) has become known as the third gasotransmitter, following nitric oxide and carbon monoxide, and the body of literature about its roles in all cells and tissues continues to expand [1,2,3]. One interesting therapeutic application of H_2_S is its use in kidney graft preservation to improve renal transplant outcomes. Renal transplantation is the preferred treatment for end stage renal disease, because it enhances the quality of life and survival compared to hemodialysis [4]. The demand for transplantable kidneys, however, outweighs the supply. As a result, kidneys obtained via donation after cardiac death (DCD) are being used more often in transplant centers across the globe [5]. DCD kidneys typically exhibit higher rates of delayed graft function (DGF) and poor long-term survival [6,7]. This is due to the greater ischemic injury experienced by DCD grafts compared to living donor organs. Kidneys from living donors experience some cold ischemic injury from the time of harvest to reimplantation. However, DCD kidneys experience prolonged periods of warm ischemia, as cardiac death occurs, and cold ischemia, during organ preservation. This leads to more severe ischemia–reperfusion injury (IRI) upon reimplantation, which contributes to DGF and other poor outcomes exhibited by DCD kidneys [8].

Organ preservation is a critical phase in terms of mitigating IRI. The clinical standard of care for renal graft preservation is static cold storage (SCS), where the organ is placed on ice (~4 °C) in a bag with preservation solution. The alternative is hypothermic machine perfusion (HMP), where the organ is perfused with cold preservation solution. While this approach improves the distribution of nutrients and improves DCD graft outcomes compared to SCS [9,10], it is used less frequently due to its higher cost and logistical challenges [11,12]. Importantly, both approaches are limited by the shutdown of cellular metabolism inherent to cold preservation, which causes renal injury while improving survival in cold conditions. Longer cold ischemia times are linked to poorer DCD graft outcomes, including higher rates of DGF [13]. As a result, therapeutic strategies that mitigate cold IRI are of interest. One therapeutic approach is supplementing preservation solutions with H_2_S due to its vasodilatory, anti-apoptotic and other cytoprotective effects during IRI [14,15,16]. We have previously shown that supplementing preservation solutions with H_2_S, especially mitochondria-targeted H_2_S donor AP39, during SCS improves murine renal graft outcomes [15,16]. However, the context of cold preservation remains. Thus, alternative approaches that facilitate kidney preservation at higher temperatures (20–37 °C) are also of interest [17].

Normothermic machine perfusion, which involves ex vivo perfusion of the kidney at 37 °C with blood-based or acellular solutions, has been shown to improve renal graft outcomes compared to SCS and HMP in several porcine DCD renal transplantation studies [18,19]. In a 2011 pilot surgical case, it was reported that a short duration of this approach after SCS improved the clinical post-transplant outcomes compared to SCS alone [20]. Additionally, several ongoing clinical trials are looking to validate and establish a clinical protocol for normothermic preservation. However, in moving away from cold preservation, research into subnormothermic preservation at different temperatures (15–35 °C) has also gained momentum. In 2014, Hoyer et al. reported that, compared to SCS and HMP, the subnormothermic machine perfusion of DCD porcine kidneys at 20 °C improved blood flow, output and creatinine clearance during reperfusion with autologous blood [21]. Our group has previously shown that adding AP39 to the University of Wisconsin (UW) solution, a preservation solution that is routinely used for SCS, made it viable for subnormothermic preservation at 21 °C [22]. We also showed that subnormothermic perfusion at 21 °C improved DCD porcine renal graft outcomes compared to SCS, subnormothermic perfusion at 15 °C and normothermic perfusion at 37 °C [23]. In this study, we investigate whether subnormothermic perfusion at 21 °C with H_2_S donor AP39 improves DCD porcine renal graft outcomes compared to SCS and subnormothermic perfusion without AP39, using ex vivo pulsatile perfusion for preservation and reperfusion.

## 2. Materials and Methods

### 2.1. Animal Care and Surgery

Ten Yorkshire pigs (60–70 kg), purchased from a regional farm, were tranquilized and routinely prepped for surgery. A midline incision was used to expose the kidneys (*n* = 20). The renal pedicles were clamped in situ for 30 min to induce warm ischemia, to mimic DCD injury, following an intravenous infusion of 10,000 units of heparin. By completely stopping the renal blood flow, we replicated an extreme clinical DCD scenario where there was no oxygen being supplied to the kidneys. This approach has been used in many other studies within this field [18,21]. The ureters and arteries were cannulated to facilitate ex vivo perfusion and urine collection. Subsequently, both kidneys were nephrectomized, and the donor animal was euthanized. Autologous blood used for perfusion was collected via cannulation of the inferior vena cava before and after clamping the renal pedicles [23]. Blood collected prior to clamping is referred to as non-stressed blood, because there is no interruption of the blood flow. However, blood collected after clamping is referred to as stressed blood due to the buildup of metabolites and signaling molecules resulting from the lack of blood flow through the kidneys. Surgeries were performed by transplant fellows at University Hospital, London, ON, Canada. All procedures were approved by the University of Western Ontario’s Animal Use Committee.

### 2.2. Treatments and Ex Vivo Perfusion Setup

**Figure 1:**
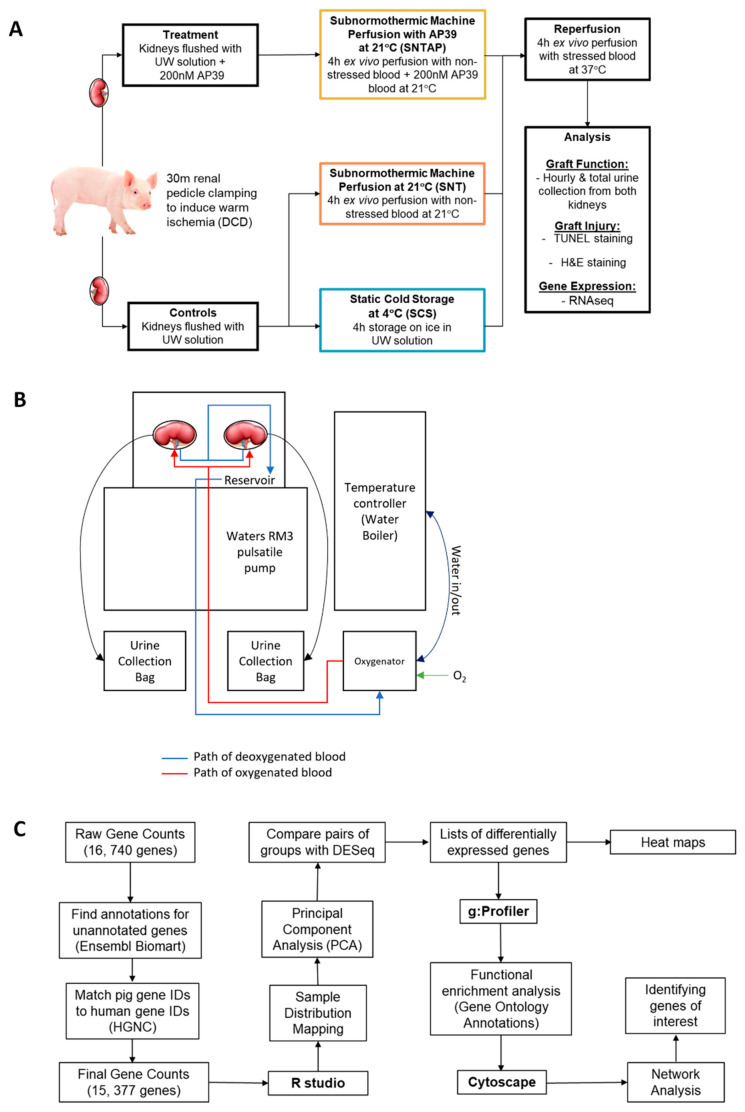
Summary of the methods. (**A**) Overview of the experimental design. (**B**) Schematic of the ex vivo perfusion setup used for porcine kidney perfusion and reperfusion. (**C**) RNAseq analysis workflow.

The kidneys were assigned to one of three preservation treatments (Figure 1A). The first group of kidneys was flushed with and stored in UW solution on ice for 4 h (SCS), which reflects the clinical standard of care. The second group of kidneys was flushed with UW solution and treated to 4 h of subnormothermic perfusion at 21 °C with non-stressed blood (SNT). The third group of kidneys was flushed with UW solution supplemented with 200 nM AP39 and treated to 4 h of subnormothermic perfusion at 21 °C with non-stressed blood supplemented with 200 nM AP39 (SNTAP). After the 4 h preservation period, all kidneys were perfused with stressed autologous blood for 4 h at 37 °C to simulate reperfusion after renal transplantation. A total of ten pig kidney perfusion experiments were performed. In the first seven experiments, left and right kidneys were randomly assigned to either the SCS group (*n* = 7) or the SNTAP group (*n* = 7), with both kidneys connected to the same cassette during reperfusion with stressed blood. Pairs of kidneys sharing the blood reservoir was unavoidable due to the limitation of having a single pulsatile pump. In the following three experiments, both the left and right kidneys were assigned to the SNT group (*n* = 6), as this was an additional control group to represent the effects of subnormothermic perfusion without AP39. The dose of AP39 was chosen based on a previous in vivo murine transplantation study by our group [10]. The kidneys were perfused using an ex vivo pulsatile perfusion apparatus (Figure 1B), which was identical to the setup used in a previous study by our center [23]. Through adjusting the flow, the mean perfusion pressure was maintained at 60 mmHg after an initial 5-min period of gradual increase. The blood used in the perfusion circuits was oxygenated using an external oxygen gas supply, and no supplemental nutrients, vasodilators or diuretics were added at any point. This was done to evaluate the effect of subnormothermic perfusion with and without AP39 compared to SCS in a simplistic manner, without any confounding variables. The urine output was recorded hourly, and PlasmaLyte solution (Baxter, Deerfield, IL, USA) was used to compensate for the fluid volume loss. Kidney samples were collected after reperfusion with stressed blood, and kidneys were bivalved sagittally. One half was stored at −80 °C for the RNA sequencing (RNAseq) analysis, and the other half was stored in formalin for histopathological analyses.

### 2.3. AP39

AP39, synthesized in-house by Prof. Whiteman [24] (Exeter, UK), was dissolved in dimethyl sulfoxide to achieve a 1 mM stock concentration. Two hundred microliters of the stock was added to 1 L bags of preservation solution used for flushing, and blood used for perfusion, to achieve a treatment concentration of 200 nM AP39 [15].

### 2.4. Histopathology Imaging and Quantification

The formalin-fixed kidney sections, including cortex and medulla, were stained with Terminal deoxynucleotidyl transferase dUTP nick end labeling (TUNEL), along with Hematoxylin and Eosin (H&E), to determine the level of apoptosis and acute tubular necrosis (ATN), respectively. TUNEL and H&E imaging was done using the Nikon Instruments Eclipse 90i digital microscope at 10× magnification (Nikon Instruments, Melville, NY, USA). To quantify the apoptotic injury, 10 random images of TUNEL staining were captured per sample. The images were run through ImageJ v.1.50 (National Institute of Health, Bethesda, MD, USA) to determine the %TUNEL+ area, the ratio of the brown tubular area (TUNEL+) and the total tubular area. To quantify the ATN, H&E slides were scored for ATN by a blinded renal pathologist, as per the following scheme: 1 = <11%, 2 = 11–24%, 3 = 25–45%, 4 = 46–75% and 5 = >75%.

### 2.5. Statistical Analysis

GraphPad Prism 8 was used to create graphs and conduct statistical analyses. Student’s *t*-test or one-way ANOVA followed by Tukey’s post-hoc test were used for comparisons of two or three experimental groups, respectively. Statistical significance was accepted at *p* < 0.05.

### 2.6. RNASeq

Total RNA was extracted from frozen renal cortical tissues using RNEasy kits (Qiagen, Toronto, ON, Canada) following the vendor’s protocols. Total RNA samples were quantified using NanoDrop (Thermo Fisher Scientific, Waltham, MA, USA), and the quality was assessed using the Agilent 2100 Bioanalyzer (Agilent Technologies Inc., Mississauga, ON, USA) and the RNA 6000 Nano kit (Caliper Life Sciences, Hanover, MD, USA). They were then processed using the Vazyme VAHTS Total RNA-seq (H/M/R) Library Prep Kit for Illumina (Vazyme, Nanjing, Jiangsu, China), which includes rRNA reduction. Three samples (*n* = 3) from each experimental group and one control sample from a baseline porcine kidney with no DCD injury (CTR, *n* = 1) were sequenced at the London Regional Genomics Centre (Robarts Research Institute, London, ON, Canada) using Illumina NextSeq 500 (Illumina Inc., San Diego, CA, USA).

Briefly, samples were rRNA-depleted, then fragmented. cDNA was synthesized, indexed, cleaned up and amplified via PCR. Libraries were then equimolarly pooled into one library, and the size distribution was assessed on an Agilent High-Sensitivity DNA Bioanalyzer chip and quantified using the Qubit 2.0 Fluorimeter (Thermo Fisher Scientific, Waltham, MA, USA). The library was sequenced on an Illumina NextSeq 500 as a single end run, 1 × 76 bp, using a High Output v2 kit (75 cycles). Fastq data files were downloaded from BaseSpace and analyzed using Partek Flow (Partek, Chesterfield, MO, USA). After importation, data was aligned to the Sus scrofa genome using STAR 2.6.1d and annotated using Ensembl v 97.

The resulting raw gene counts were analyzed using a workflow summarized in Figure 1C. A final gene counts file was prepared after removing the genes that could not be annotated using Ensembl Biomart and genes that did not have matching human gene IDs approved by the HUGO Gene Nomenclature Committee, which is necessary for functional enrichment analysis. Using RStudio v. 3.6.1 (Boston, MA, USA; session info in Appendix A), sample distribution mapping and principal component analysis were performed to visualize the difference in the expression profiles of the samples in each group. The DESeq2 package was used to identify the differentially expressed genes for the following comparisons: SNTAP vs. SCS and SNTAP vs. SNT (alpha = 0.05). Based on the protocol detailed by Reimand et al. [25], Gene Ontology (GO) annotations were found for the differentially expressed genes using g:Profiler. These annotations were analyzed using Cytoscape v.3.7.2 to develop network enrichment maps, which facilitated the identification of certain genes and pathways of interest for both comparisons.

## 3. Results

### 3.1. Subnormothermic Perfusion with AP39-Supplemented Blood Improves Urine Output and Reduces Tissue Injury Compared to Static Cold Storage and Subnormothermic Perfusion without AP39

**Figure 2:**
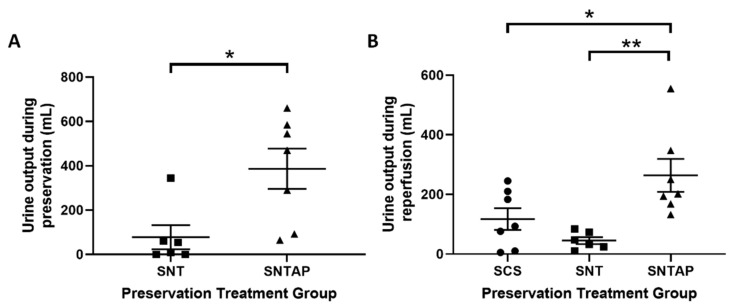
Subnormothermic perfusion with AP39 improves urine output during preservation and reperfusion. Porcine kidneys were nephrectomized after 30 min of clamping to mimic donation after cardiac death (DCD) injury and subjected to various preservation treatments for 4 h. This was followed by 4 h of reperfusion with stressed autologous blood. Urine output was collected during both perfusion periods for all groups, except for the static cold storage group, where kidneys were on ice during the 4 h of preservation. (**A**) Total urine output (mL) collected during 4 h of preservation perfusion. (**B**) Total urine output (mL) collected during 4 h of reperfusion with stressed blood. Lines represent mean ± SEM. Values in (**A**) were compared using a student’s unpaired *t*-test, and values in (**B**) were compared using one-way ANOVA followed by Tukey’s post-hoc test. * *p* < 0.05 and ** *p* < 0.01. Circles represent values for static cold storage (SCS, *n* = 7). Squares represent values for subnormothermic perfusion (SNT, *n* = 6). Triangles represent values for subnormothermic perfusion with 200 nM AP39 (SNTAP, *n* = 7).

**Figure 3:**
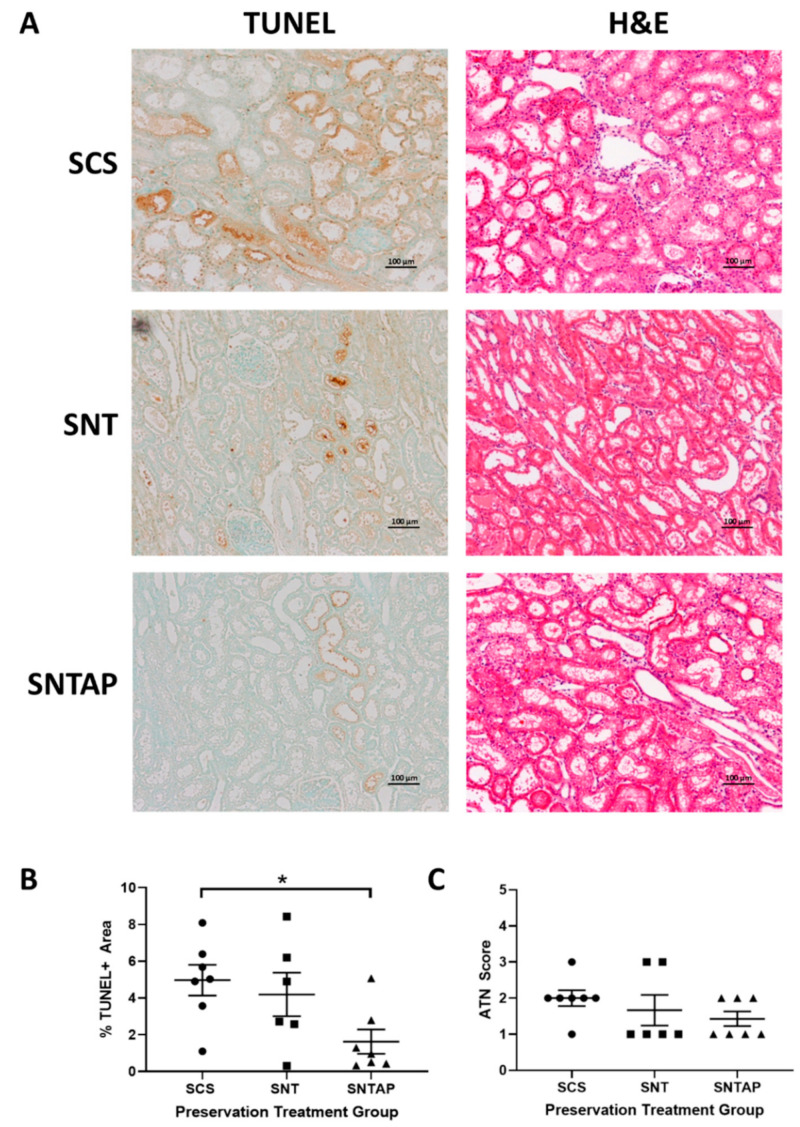
Subnormothermic perfusion with AP39 reduces tissue apoptosis and acute tubular necrosis. (**A**) Representative Terminal deoxynucleotidyl transferase dUTP nick end labeling (TUNEL) and Hematoxylin and Eosin (H&E) images of formalin-fixed DCD kidney sections after 4 h of preservation treatment and 4 h of reperfusion. Images were taken at 10× magnification (scale bar = 100 µm). (**B**) Mean %TUNEL+ area, as determined by ImageJ, using a ratio of the TUNEL+ area (brown) to total tubular area. Each individual data point represents the mean %TUNEL+ area of 10 random fields of view of one porcine kidney sample. (**C**) Acute tubular necrosis (ATN) scores based on H&E staining. Each individual data point represents the score assigned to one porcine kidney sample. Lines in (**B**,**C**) represent the means ± SEM. Values in (**B**,**C**) were compared using one-way ANOVA followed by Tukey’s post-hoc test. * *p* < 0.05. Circles represent values for static cold storage (SCS, *n* = 7). Squares represent values for subnormothermic perfusion (SNT, *n* = 6). Triangles represent values for subnormothermic perfusion with 200 nM AP39 (SNTAP, *n* = 7).

To investigate whether subnormothermic perfusion with AP39-supplemented blood can improve ex vivo DCD renal graft function, porcine kidneys were assigned to four h of cold storage or subnormothermic perfusion with non-stressed blood at 21 °C, with or without AP39. During preservation, DCD kidneys perfused with AP39-supplemented blood (SNTAP group) exhibited significantly higher (*p* < 0.05) urine output than the kidneys perfused with blood on their own (SNT group) (Figure 2A). No urine output was recorded for the static cold storage (SCS group), as those kidneys were placed on ice. The preservation period was followed by four h of reperfusion with stressed blood at 37 °C. During reperfusion, the SNTAP group also exhibited significantly higher urine output than the SCS group (*p* < 0.05) and the SNT group (*p* < 0.01). No significant difference in urine output was observed between the SCS and SNT groups (Figure 2B). Following reperfusion, the kidneys were sagittally bivalved, and formalin-fixed sections were stained with TUNEL and H&E to determine apoptotic injury and ATN, respectively (Figure 3A). The SNTAP group exhibited the lowest mean %TUNEL+ area and mean ATN score (Figure 3B,C). While no statistically significant differences were found amongst the mean ATN scores (Figure 3C), the SNTAP group exhibited significantly lower mean %TUNEL+ area than the SCS group (*p* < 0.05), indicating significantly lower apoptotic injury (Figure 3B).

### 3.2. Adding AP39 to Blood During Subnormothermic Perfusion Leads to Differential Pro--Survival Gene Expression Patterns Compared to Static Cold Storage and Subnormothermic Perfusion without AP39

**Figure 4:**
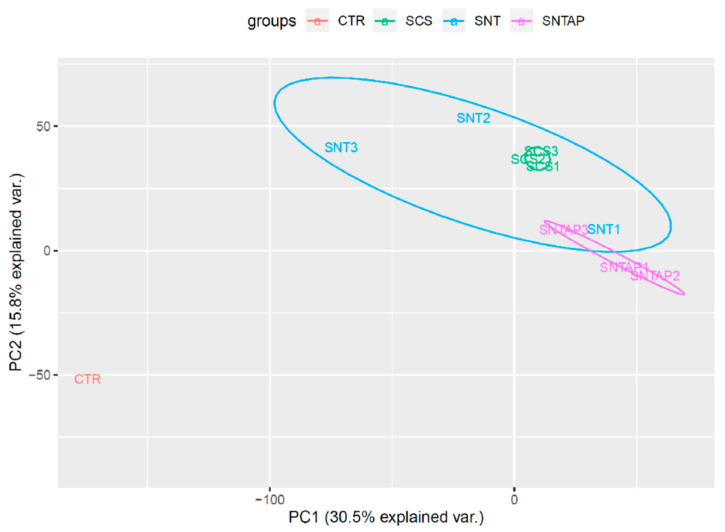
Principal component analysis of r-log normalized gene counts for all porcine kidney samples. Frozen renal cortical sections were used for the RNAseq analysis. RStudio was used to perform the principal component analysis with the full set of r-log normalized gene counts for all samples and to visualize the clustering of samples by group. CTR, control baseline kidney with no DCD injury or preservation treatment (*n* = 1). SCS, static cold storage (*n* = 3). SNT, subnormothermic perfusion (*n* = 3). SNTAP, subnormothermic perfusion with 200 nM AP39 (*n* = 3). Numbers following group names denote individual samples.

**Figure 5:**
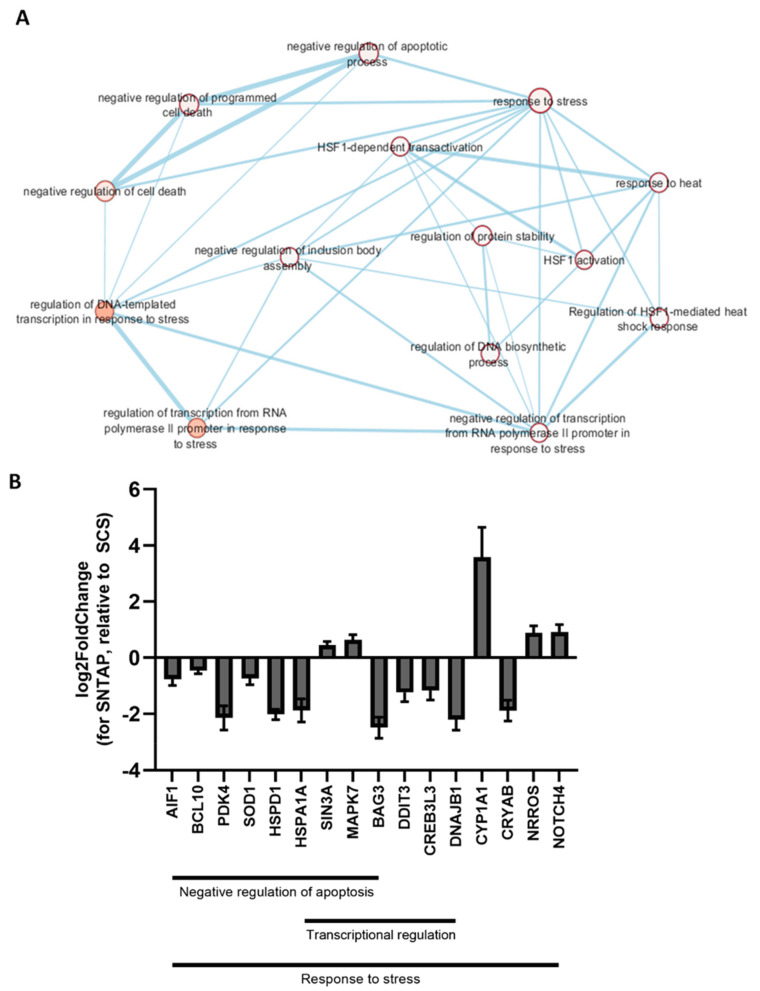
Gene expression analysis after preservation using subnormothermic perfusion with AP39 vs. static cold storage. Frozen renal cortical sections (*n* = 3 for each group) were used for the RNAseq analysis. Differentially expressed genes were identified using RStudio (DESeq2; alpha = 0.05). Gene Ontology (GO) annotations were found for those genes using g:Profiler, and the network enrichment analysis was performed using Cytoscape. (**A**) Network enrichment map showing certain nodes of interest (FDR Q value < 1.0 and Jaccard Overlap combined coefficient > 0.375 with a combined constant = 0.5). (**B**) Gene expression values (log2 fold change ± SEM, determined using RStudio) of certain genes of interest chosen from three nodes of interest from the enrichment map in (**A**)—response to stress, negative regulation of the apoptotic process and regulation of the DNA-templated transcription in response to stress.

**Figure 6:**
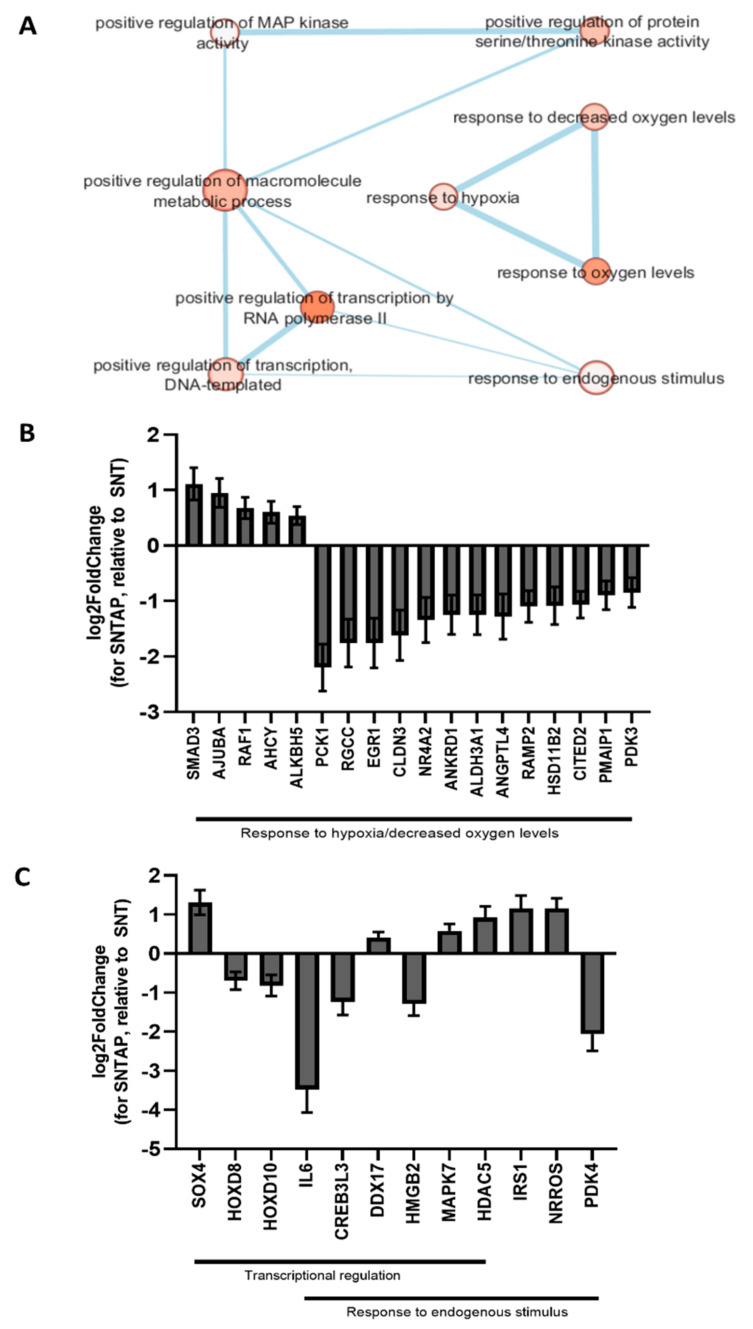
Gene expression analysis after preservation using subnormothermic perfusion with or without AP39. Frozen renal cortical sections (*n* = 3 for each group) were used for the RNAseq analysis. Differentially expressed genes were identified using RStudio (DESeq2, alpha = 0.05). GO annotations were found for those genes using g:Profiler, and the network enrichment analysis was performed using Cytoscape. (**A**) Network enrichment map showing certain nodes of interest (FDR Q value < 1.0 and Jaccard Overlap combined coefficient > 0.375 with a combined constant = 0.5). (**B**) Gene expression values (log2 fold change ± SEM, determined using RStudio) of all the genes in the three connected nodes from the enrichment map in (**A**)—response to oxygen levels, hypoxia and decreased oxygen levels. (**C**) Gene expression values (log2 fold change ± SEM, determined using RStudio) of certain genes of interest from two nodes of interest from the enrichment map in (**A**)—regulation of the DNA-templated transcription and response to the endogenous stimulus.

To investigate the effect of AP39 on gene expression in this study, RNAseq was performed on frozen porcine kidney sections collected after reperfusion. Using RStudio, a principal component analysis was performed to visualize the kidney samples in all groups (Figure 4). Using the DESeq2 package in RStudio, it was determined that 214 genes were differentially expressed in the SNTAP group vs. the SCS group (alpha = 0.05, Figure S1 in the Supplementary Materials, which is available online). Through the network enrichment analysis, clusters of interest were identified based on GO annotations for the 214 genes, including the response to heat, response to stress, regulation of transcription and negative regulation of cell death (Figure 5A). Relative to the SCS group, the SNTAP group exhibited downregulation of pro-apoptotic (BCL10) and heat shock response (HSPD1 and HSPA1A) genes, along with regulators of those pathways (BAG3 and DDIT3). Additionally, proliferation (MAPK7) and oxidative stress response (NRROS) genes were upregulated in the SNTAP group (Figure 5B). Next, it was determined that 614 genes were differentially expressed in the SNTAP group vs. the SNT group (alpha = 0.05). Clusters of interest were identified based on GO annotations, including the response to hypoxia, regulation of transcription and response to endogenous stimulus (Figure 6A). Relative to the SNT group, several genes associated with the HIF-1α-mediated hypoxia response pathway (EGR1, PCK1, PDK3 and RGCC) were downregulated in the SNTAP group. The expression of genes mediating the transforming growth factor beta (TGF-β) pathway (SMAD3 and NRROS) and HIF-1α degradation (AJUBA) was upregulated in the SNTAP group (Figure 6B). Additionally, the downregulation of proinflammatory (IL6 and HMGB2) and pro-cell death (HOXD8, HOXD10) genes was observed in the SNTAP group, along with upregulation of proliferation (MAPK7) and oxidative stress response (NRROS) genes (Figure 6C).

## 4. Discussion

This study shows that subnormothermic perfusion of DCD pig kidneys with AP39-supplemented blood (SNTAP) improves the urine output, reduces tissue injury and leads to differential pro-survival gene expression compared to static cold storage (SCS) and subnormothermic perfusion with blood alone (SNT) in our ex vivo model of preservation (four hours) and reperfusion (four hours).

The SNTAP group displayed a significantly higher urine output than the SNT group during preservation and both the SNT and SCS groups during reperfusion. This is likely due to an increase in vasodilation leading to greater renal blood flow (RBF) and subsequent diuresis. Vasoconstriction is a critical consequence of warm ischemic injury during DCD organ procurement and cold ischemic injury during SCS or HMP [26]. H_2_S has been shown to enhance vasodilation and RBF in several studies. Xia et al. showed that the infusion of NaHS in the intrarenal arteries of rats enhanced RBF and the glomerular filtration rate [27]. Additionally, using a porcine model of renal transplantation, Hosgood and Nicholson showed that the infusion of H_2_S ten minutes prior to and after reperfusion improves RBF and renal function [28]. Previous studies have also shown that H_2_S inhibits platelet aggregation in vitro and in vivo [29,30], which could contribute to increased blood flow. Although AP39 has not been directly linked to RBF or platelet aggregation to date, our RNAseq results (discussed below) suggest a potential vasodilatory mechanism that may contribute to the significantly higher urine output of the SNTAP group.

The SNTAP group also exhibited significantly lower tissue apoptosis than the SCS group, based on TUNEL staining. This finding was supported by the downregulation of pro-apoptotic genes in the SNTAP group relative to the SCS group. Several studies have reported that AP39 reduces renal apoptosis [15,31,32], and it has been shown to downregulate Bax [32], a pro-apoptotic gene in the intrinsic apoptotic pathway. This study shows, for the first time, that AP39 may be exerting anti-apoptotic effects through downregulating BCL10 [33,34], a pro-apoptotic regulatory gene, and DDIT3 [35], a transcriptional regulator of endoplasmic reticulum stress-induced apoptosis. Notably, the expression of heat shock proteins (HSPD1 and HSPA1A) and BAG3, an HSP cochaperone [36], was downregulated in the SNTAP group. Seeing as these factors normally promote cell survival during heat-induced stress [37,38], it is counterintuitive that they are downregulated in the SNTAP group (21 °C) compared to the SCS group (4 °C). However, the resulting anti-survival effects are likely counteracted by the downregulation of apoptotic genes and upregulation of MAPK7, a key mediator of cell proliferation and cell survival [39].

Interestingly, several genes associated with the HIF1α-mediated hypoxia response, which contributes to acute kidney injury and renal IRI [40], were downregulated in the SNTAP group relative to the SNT group. The downregulation of PCK1 [41] and PDK3, metabolic targets activated by HIF1α [42], may help with managing the metabolic demand at 21 °C that is not being met with metabolites, thereby mitigating tissue injury. The downregulation of RGCC, a negative regulator of HIF1α-mediated angiogenesis [43], and EGR1, a negative coregulator of erythropoietin-receptor expression [44], likely promotes vascularization. This may contribute to the improved urine output of the SNTAP group through improving the RBF. The upregulation of AJUBA, a mediator of HIF1α degradation [45], likely helps to balance the positive and negative effects of HIF1α and other genes associated with it. Additionally, the SNTAP group exhibited downregulation of the pro-apoptotic genes (HOXD8 and HOXD10) [46,47], which could further explain the lower tissue apoptosis in the SNTAP group.

The SNTAP group also exhibited upregulation of MAPK7 and NRROS compared to both the SCS and SNT groups. AP39 has been shown to reduce reactive oxygen species (ROS) levels in vitro [15,32]. The downregulation of NRROS, which negatively regulates ROS production [48] and activates TGF-β1 [49,50], could underlie the antioxidant and protective responses observed by previous studies on AP39. Additionally, the downregulation of IL6 was observed in the SNTAP group relative to the SNT group, which matches the findings of a previous study looking at the effect of AP39 in myocardial IRI [32]. IL6 is a key proinflammatory cytokine in the progression of acute kidney injury [51], and its downregulation by AP39 may contribute to the improved outcomes of the SNTAP group compared to the SNT group.

There are two limitations to the perfusion model used in this study. Firstly, the perfusion times used are relatively short. However, the four-hour duration was deemed appropriate, because this was the first time that our novel approach towards organ preservation was attempted on large mammalian kidneys, and hence, we wanted to prove its feasibility. Further testing using longer periods of preservation and reperfusion will be necessary for clinical translation. Secondly, the limitation of having only one organ perfusion pump due to financial constraints required us to place the two kidneys on the same circuit during the reperfusion phase; however, the urine output from each kidney was collected separately. Perfusing both kidneys on separate pumps would have prevented their metabolites from pooling into a shared blood reservoir. Despite this limitation, the SNTAP group exhibited significantly higher urine output during reperfusion and a significantly lower TUNEL score than the SCS group. This suggests that if we had reperfused the kidneys on two separate pumps, our positive findings would have been accentuated to a greater extent, which further supports the use of our novel approach.

While the number of samples we could perform RNAseq testing on was limited by the cost, the consistency in gene expression across the samples in most groups facilitated a reliable analysis within the constraints of this limitation. Additionally, the findings of our study were strengthened by the approach of clamping the renal pedicle to mimic a DCD injury. Inducing the complete cessation of the RBF via clamping mimics an extreme clinical DCD scenario where no oxygen is supplied to the kidneys. Thus, the positive outcomes observed in this study are more likely to translate to and be accentuated in real clinical DCD scenarios where there is a more gradual cessation of blood flow as cardiac death occurs. Evidently, further in vitro research is needed to confirm the mechanisms underlying AP39′s protective effects. Future studies using in vivo models of renal transplantation and clinically approved H_2_S donors are also needed to support the findings of this study.

In conclusion, this study demonstrates that subnormothermic perfusion with AP39-supplemented blood can improve ex vivo DCD porcine renal graft function. We postulate that the subnormothermic temperature allows for maintenance of the vasculature, unlike static cold storage, with a metabolic demand that is lower than normal due to the deviation from the physiological temperature (37 °C). The presence of H_2_S helps mitigate some of the negative consequences of being unable to meet the metabolic demand at 21 °C in a nutrient-deficient environment, such as inflammation, ROS and apoptosis. Overall, this study adds to the growing body of literature that supports the use of subnormothermic temperatures and H_2_S in organ preservation to improve DCD renal graft outcomes. These strategies could facilitate the use of more marginal grafts, which could increase the pool of transplantable organs.

## 5. Patent

The data reported in this study is a part of a US patent application (serial no. 17/127,965 entitled “Method and Compositions for Protecting Tissue”) that is pending approval. Additionally, Prof. Matthew Whiteman and the University of Exeter have intellectual property (patent filings) related to hydrogen sulfide delivery molecules and their therapeutic use.

## Data Availability

The data presented in this study are available on request from the corresponding author.

## Supplementary Materials

The following are available online at https://www.mdpi.com/2218-273X/11/3/446/s1: Figure S1: Heatmap of the differential gene expression analysis after preservation using subnormothermic perfusion with AP39 vs. static cold storage. Porcine kidneys were nephrectomized after 30 min of clamping to mimic DCD injury and subjected to subnormothermic perfusion with AP39 (SNTAP) or static cold storage (SCS) for 4 h. This was followed by 4 h of reperfusion with stressed autologous blood. Frozen renal cortical sections (*n* = 3 for each group) were used for the RNAseq analysis. Differentially expressed genes were identified using RStudio (DESeq2, alpha = 0.05), and a heap map was generated. CTR, control baseline kidney with no DCD injury or preservation treatment (*n* = 1).

## Abbreviations

AJUBA	Lim-domain containing protein AJUBA
BAG3	Bcl2-associated anthanogene (BAG) family chaperone regulator 3
BCL10	B-cell lymphoma/leukemia 10
DDIT3	DNA damage-inducible transcript 3
EGR1	Early growth response protein 1
HOXD8/10	Homeobox protein Hox-D8/10
HSPA1A	Heat shock 70kDa protein 1A
HSPD1	Heat shock protein 60kDa, mitochondrial
IL6	Interleukin 6
MAPK7	Mitogen-activated protein kinase 7
NRROS	Negative regulator of reactive oxygen species
PCK1	Phosphoenolpyruvate carboxykinase, cytosolic
PDK3	Pyruvate dehydrogenase kinase, mitochondrial
RGCC	Regulator of the cell cycle
SMAD3	Mothers against decapentaplegic homolog 3
TGF-β	Transforming growth factor β

## Appendix A

RStudio session info:

R version 3.6.1 (22 March 2020)

Platform: x86_64-w64-mingw32/x64 (64-bit)

Running under: Windows 10 x64 (build 17763)

Matrix products: default

locale:

[1] LC_COLLATE=English_Canada.1252 LC_CTYPE=English_Canada.1252

[3] LC_MONETARY=English_Canada.1252 LC_NUMERIC=C

[5] LC_TIME=English_Canada.1252

attached base packages:

[1] grid parallel stats4 stats graphics grDevices utils datasets [9] methods base

other attached packages:

[1] DEGreport_1.22.0 readxl_1.3.1 readr_1.3.1

[4] vsn_3.54.0 hexbin_1.28.0 ggbiplot_0.55

[7] scales_1.0.0 plyr_1.8.4 ggplot2_3.2.1

[10] pheatmap_1.0.12 RColorBrewer_1.1-2 IHW_1.14.0

[13] DESeq2_1.26.0 SummarizedExperiment_1.16.0 DelayedArray_0.12.0

[16] BiocParallel_1.20.0 matrixStats_0.55.0 Biobase_2.46.0

[19] GenomicRanges_1.38.0 GenomeInfoDb_1.22.0 IRanges_2.20.0

[22] S4Vectors_0.24.0 BiocGenerics_0.32.0

loaded via a namespace (and not attached):

[1] colorspace_1.4-1 rjson_0.2.20 ellipsis_0.3.0

[4] circlize_0.4.8 htmlTable_1.13.2 XVector_0.26.0

[7] ggdendro_0.1-20 GlobalOptions_0.1.1 base64enc_0.1-3

[10] clue_0.3-57 rstudioapi_0.10 affyio_1.56.0

[13] ggrepel_0.8.1 bit64_0.9-7 AnnotationDbi_1.48.0

[16] splines_3.6.1 logging_0.10-108 mnormt_1.5-5

[19] geneplotter_1.64.0 knitr_1.25 zeallot_0.1.0

[22] Formula_1.2-3 Nozzle.R1_1.1-1 broom_0.5.2

[25] annotate_1.64.0 cluster_2.1.0 png_0.1-7

[28] BiocManager_1.30.9 compiler_3.6.1 backports_1.1.5

[31] assertthat_0.2.1 Matrix_1.2-17 lazyeval_0.2.2

[34] limma_3.42.0 lasso2_1.2-20 acepack_1.4.1

[37] htmltools_0.4.0 tools_3.6.1 glue_1.3.1

[40] gtable_0.3.0 GenomeInfoDbData_1.2.2 affy_1.64.0

[43] dplyr_0.8.3 Rcpp_1.0.3 slam_0.1-46

[46] cellranger_1.1.0 vctrs_0.2.0 nlme_3.1-140

[49] preprocessCore_1.48.0 psych_1.8.12 xfun_0.10

[52] stringr_1.4.0 lifecycle_0.1.0 XML_3.98-1.20

[55] edgeR_3.28.0 MASS_7.3-51.4 zlibbioc_1.32.0

[58] hms_0.5.2 ComplexHeatmap_2.2.0 memoise_1.1.0

[61] gridExtra_2.3 rpart_4.1-15 reshape_0.8.8

[64] latticeExtra_0.6-28 stringi_1.4.3 RSQLite_2.1.2

[67] genefilter_1.68.0 checkmate_1.9.4 shape_1.4.4

[70] rlang_0.4.1 pkgconfig_2.0.3 bitops_1.0-6

[73] lattice_0.20-38 lpsymphony_1.14.0 purrr_0.3.3

[76] htmlwidgets_1.5.1 labeling_0.3 cowplot_1.0.0

[79] tidyselect_0.2.5 bit_1.1-14 magrittr_1.5

[82] R6_2.4.0 generics_0.0.2 Hmisc_4.3-0

[85] DBI_1.0.0 pillar_1.4.2 foreign_0.8-71

[88] withr_2.1.2 survival_2.44-1.1 RCurl_1.95-4.12

[91] nnet_7.3-12 tibble_2.1.3 crayon_1.3.4

[94] fdrtool_1.2.15 GetoptLong_0.1.7 locfit_1.5-9.1

[97] data.table_1.12.6 blob_1.2.0 ConsensusClusterPlus_1.50.0

[100] digest_0.6.22 xtable_1.8-4 tidyr_1.0.0

[103] munsell_0.5.0
