# Supplementary figures and images for: Subnormothermic Perfusion with H2S Donor AP39 Improves DCD Porcine Renal Graft Outcomes in an Ex Vivo Model of Kidney Preservation and Reperfusion
Source: Biomolecules. 2021 Mar 17;11(3):446. doi: 10.3390/biom11030446 (PMC8002411; doi:10.3390/biom11030446)

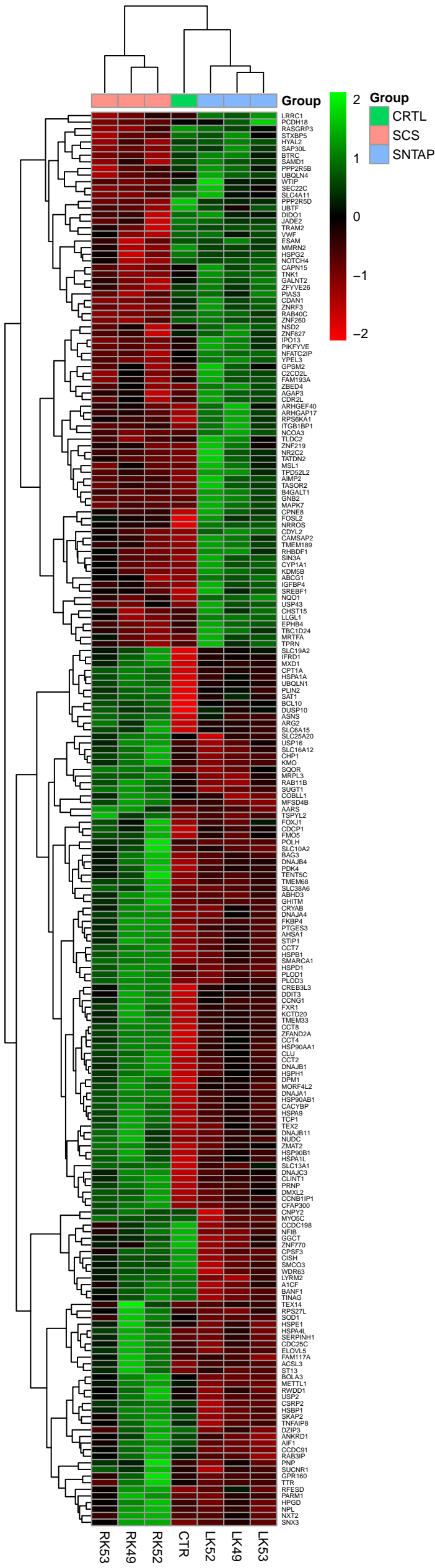

Supplement: Supplementary file 1 [file biomolecules-11-00446-s001.pdf]
